# Mechanical Properties of Organelles Driven by Microtubule-Dependent Molecular Motors in Living Cells

**DOI:** 10.1371/journal.pone.0018332

**Published:** 2011-04-01

**Authors:** Luciana Bruno, Marcelo Salierno, Diana E. Wetzler, Marcelo A. Despósito, Valeria Levi

**Affiliations:** 1 Departamento de Física, Facultad de Ciencias Exactas y Naturales, Universidad de Buenos Aires, Pabellón 1, Ciudad Universitaria, Ciudad de Buenos Aires, Argentina; 2 Departamento de Química Biológica, Facultad de Ciencias Exactas y Naturales, Universidad de Buenos Aires, Ciudad Universitaria, Ciudad de Buenos Aires, Argentina; University of Geveva, Switzerland

## Abstract

The organization of the cytoplasm is regulated by molecular motors which transport organelles and other cargoes along cytoskeleton tracks. Melanophores have pigment organelles or melanosomes that move along microtubules toward their minus and plus end by the action of cytoplasmic dynein and kinesin-2, respectively. In this work, we used single particle tracking to characterize the mechanical properties of motor-driven organelles during transport along microtubules. We tracked organelles with high temporal and spatial resolutions and characterized their dynamics perpendicular to the cytoskeleton track. The quantitative analysis of these data showed that the dynamics is due to a spring-like interaction between melanosomes and microtubules in a viscoelastic microenvironment. A model based on a generalized Langevin equation explained these observations and predicted that the stiffness measured for the motor complex acting as a linker between organelles and microtubules is ∼ one order smaller than that determined for motor proteins in vitro. This result suggests that other biomolecules involved in the interaction between motors and organelles contribute to the mechanical properties of the motor complex. We hypothesise that the high flexibility observed for the motor linker may be required to improve the efficiency of the transport driven by multiple copies of motor molecules.

## Introduction

Molecular motors are responsible for the intracellular transport of a wide variety of components positioning them in the cytoplasm with high spatial-temporal precision. Three different classes of motors are involved in this task: dynein and kinesin, which transport cargoes toward the minus and plus ends of microtubules, respectively, and myosin, responsible for the transport along actin filaments (reviewed in [1], [2]).

One of the cellular systems widely used to study transport driven by motors are melanophore cells [3]. These cells have pigment organelles called melanosomes, which contain the black pigment melanin. Melanosomes distribute in the cells in two configurations: either aggregated in the perinuclear region or homogeneously dispersed in the cytoplasm. The transport of pigment organelles during aggregation and dispersion is regulated by signaling mechanisms initiated by the binding of specific hormones to cell surface receptors, which results in the modulation of cAMP concentrations [4], [5]. Pigment dispersion requires the plus-end directed microtubule motor kinesin-2 [6] and the actin motor myosin-V [7], whereas aggregation is powered by the minus-end directed motor cytoplasmic dynein [8].

Biophysical properties of molecular motors have been extensively studied by single molecule/particle techniques which provided extremely valuable information both in vitro [9] and in living cells [10], [11], [12], [13]. A key question for understanding motor-driven transport in living cells is how the force developed by the motor (∼1–10 pN [14], [15], [16]) is translated into cargo transport. In this sense, the stiffness of the molecular linker between the microtubule and the organelle and the properties of the organelle microenvironment might play important roles. A stiff linker determines that the motions of the motor and the organelle are highly correlated contrary to what it would be expected for a flexible linker. On the other hand, the kinetics of melanosome response to the motor stepping will be related to the rheological properties of the organelle microenvironment.

The stiffness of kinesin has been determined *in vitro* by optical trapping techniques and ranged between 0.2–0.6 pN/nm depending on the conditions of the assays [17], [18], [19], [20]. In these experiments, motors are attached to artificial cargos such as glass or polystyrene beads by different protocols which include indirect linkers such as streptavidin-biotin [21] or direct adsorption to a surface treated with a blocking protein [22]. The interactions with the surface, the linkers and/or blocking molecules may affect the properties of the motor as was proposed to explain the different behavior of heavy meromyosin when attached to surfaces with different hydrophobicities [23].

In living cells, molecular motors bind to organelles through different molecular mechanisms in which specific domains of the motor molecules and associated proteins have a key role (reviewed in [24], [25]). In the particular case of frog melanophores, it is not completely clear how motors anchor to melanosomes. It is believed that the multimeric protein complex dynactin plays an important role on attaching dynein to the organelle membranes (reviewed in [26]). Moreover, it has been shown that minus and plus end motors compete for binding to the same region of this protein complex and that impairment of the dynactin complex abolishes both plus and minus end motion of several bidirectional cargoes [27], [28]. Also, dynactin increases the processivity of kinesin-2 [29] and of cytoplasmic dynein [30]. A recent work showed that this last motor attaches to organelles even in the absence of dynactin although transport is suppressed in this condition [31]. Regardless of the exact mechanism of attachment to the membrane, it is expectable that the overall mechanical properties of the motor linker, i.e. the anchoring complex formed by molecular motors and adaptor proteins, will differ to that observed for molecular motors in *in vitro* conditions.

In this work we explore the mechanical properties of the motor linker in organelles actively transported along microtubules in living cells. With this aim, we used single particle tracking (SPT) to obtain trajectories of the organelles in processive transport along microtubules with high temporal and spatial resolutions. We quantitatively analyzed the dynamics of melanosomes perpendicular to the main transport direction. These data could be interpreted according to a model based on a generalized Langevin equation which allowed us to determined that the stiffness for both dynein and kinesin transported organelles is ∼ one order smaller than the stiffness measured for molecular motors *in vitro*. We also analyzed the motion of melanosomes in cells in which dynactin was disrupted and observed that the stiffness of the motor linker depends on dynactin integrity. This result suggests that other biomolecules involved in the interaction between motors and organelles contribute to the mechanical properties of the motor complex.

We hypothesize that flexible motor linkers might facilitate collective transport driven by multiple copies of molecular motors in living cells.

## Results and Discussion

### The dynamics of melanosomes perpendicular to the microtubule axis is due to a spring-like interaction

Melanophores were treated with latrunculin as described in Materials and Methods in order to depolymerize actin filaments. In this condition, active motion is only driven by microtubule-dependent motors. After this treatment, aggregation and dispersion of melanosomes were induced by addition of melatonin and MSH, respectively.

Movies of aggregating and dispersing cells were recorded and analyzed with a pattern-recognition algorithm [10] to obtain trajectories of melanosomes moving along microtubules. We classified the trajectories showing continuous motion either toward or away the cell nucleus according to the motor responsible for the transport i.e. cytoplasmic dynein and kinesin-2, respectively.

In order to analyze the motion of melanosomes perpendicular to the transport direction we first identified in these trajectories segments of 200 data points (0.6 s) showing a continuous, curvilinear motion. The distance traveled by melanosomes in this temporal window was <2 µm and the speed of the organelles was approximately constant with values ranging between 0.2 and 3 µm/s.

On the other hand, we imaged melanophore cells expressing EGFP-tagged XTP by confocal microscopy to characterize the curvature of microtubules. XTP is a *Xenopus* homologue of tau protein which binds to microtubules and therefore, allows their visualization. We analyzed the curvature of 2 µm long segments of microtubules (i.e. similar to the distance traveled by melanosomes in the trajectory segments analyzed before) and observed that they could be correctly described with a second-order polynomial and an average curvature of (1.8±0.6) 10^−4^ µm^−1^.

Since we did not observed significant motion of the microtubule track in the temporal window of these experiments (Text S1 and Figures S1 and S2), we considered that the mean direction of transport is given by the shape of the underlying microtubule track and calculated this direction by fitting a second order polynomial to each melanosome trajectory segments [average curvature  =  (2.2±0.7)10^−4^ µm^−1^].

**Figure 1:**
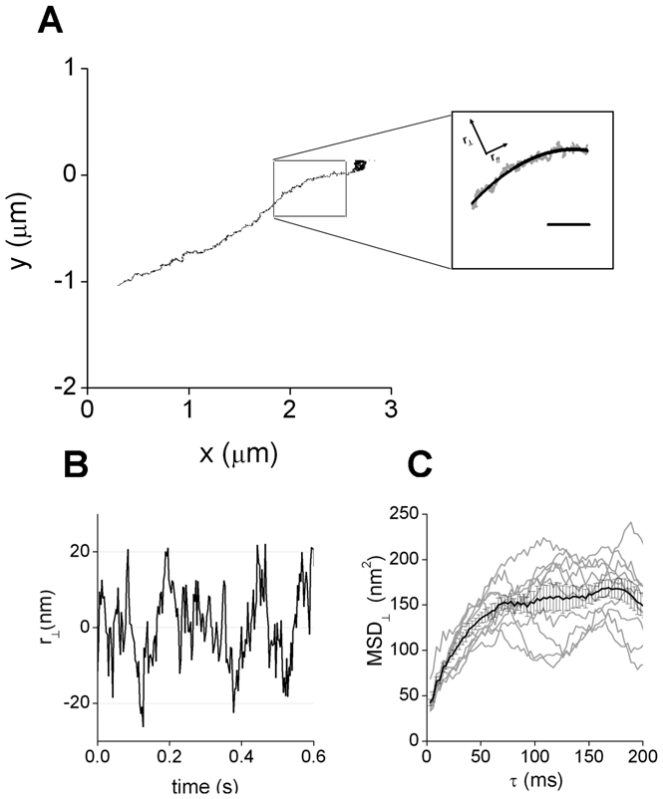
Analysis of melanosomes motion perpendicular to the transport axis. (A) Representative trajectory of a melanosome moving along a microtubule. The continuous line shows the average position calculated for the microtubule. Scale bar, 200 nm (B) Motion on the perpendicular direction obtained after analyzing the trajectory represented before (C) Mean square displacement obtained from characteristic trajectory segments for the motion perpendicular to the transport direction (gray lines). The black lines show the average and standard error calculated for each data point.

These segments were further decomposed into parallel (r_//_) and perpendicular (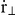) motion with respect to the transport direction axis (Figure 1). This last component was calculated as the shortest distance from each experimental data point to the microtubule track.

The mean square displacement of the motion perpendicular to the microtubule (MSD_⊥_) was obtained for each analyzed trajectory segment as
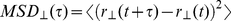
(1)where t and τ are the absolute and lag times, respectively and the brackets represents the time average.

Figure 1C shows that MSD_⊥_ increases with τ until it reaches a constant value after ∼70 ms. This behavior is characteristic of a confined motion as would be obviously expected since melanosomes do not diffuse away from the microtubule while being transported. However, the confinement can be due to different mechanisms, e.g. the elastic interaction of the melanosome with the microtubule through the molecular motor and adaptor molecules, or trapping in a crowded microenvironment. The analysis of MSD_⊥_ does not allow distinguishing one mechanism from the other.

To gain further insight into the mechanism responsible for the confinement, we calculated the particle density distribution function (PDDF) for the 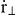 data as
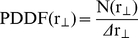
(2)where Δ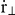 is the distribution resolution, and N(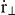) the number of particles with distances r_o_ in the range 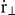
- Δ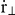/2 ≤r_o_<
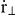
+ Δ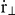/2. This distribution was normalized by PDDF(0) for nonlinear least squares regression of Boltzmann distribution functions.

Jin et al [32] showed that the particle density distribution function obtained from single particle trajectories is given by the physical mechanism responsible for the confinement. Therefore, we can extract valuable information of the confinement mechanism by analyzing the PDDF obtained for 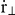.

**Figure 2:**
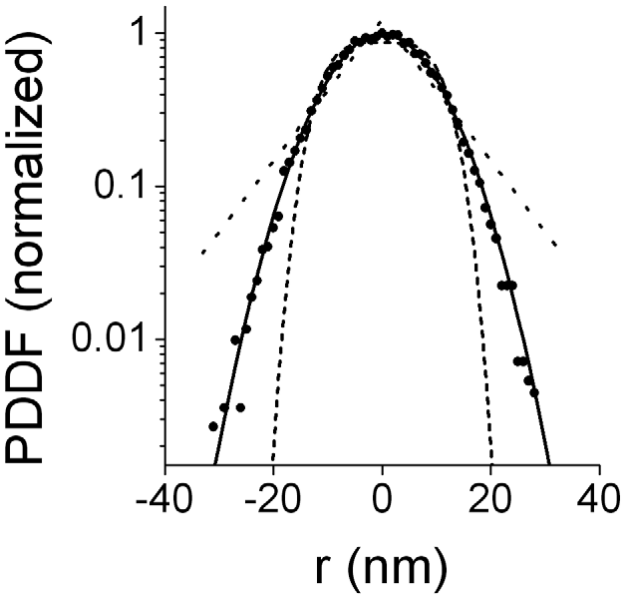
Analysis of melanosome position density distribution function. 80 segments of trajectories corresponding to kinesin-driven melanosomes in MSH-stimulated cells were analyzed as described previously to obtain 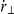. These data were used to calculate the normalized particle density distribution function PDDF(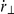)/PDDF(0) using Δ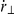 = 1 nm. The continuous black line shows the fitting of equation (3); dotted and dashed lines represent the fitting of models corresponding to cone and r^4^ potentials, respectively.

Figure 2 shows representative data of the normalized particle density distribution calculated as described above for kinesin-driven melanosomes and the results obtained by fitting these data with 3 different models of confinement [32]:

1) a *harmonic potential U(r) =  Uo r^2^*, where particles are bounded by a springlike force-producing mechanism;

2) a *cone potential U(r)  =  Uo r*, where particles are trapped by a softer potential than that of the harmonic one; and

3) a *r^4^ potential U(r)  =  Uo r^4^*, which is harder than the spring potential

where U(r) is the potential function, r is the radius from the potential origin and *Uo* is the potential strength.

As can be observed, the normalized PDDF is correctly described with a spring-like potential which is represented by the Boltzmann distribution,
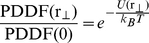
(3)where U(r) is the harmonic potential 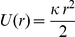, κ is the stiffness of the potential, k_B_ is the Boltzmann constant and T is the absolute temperature.

We obtained κ  = 0.0278±0.004 pN/nm by fitting equation (3) to the data showed in Figure 2. Similar results were obtained for dynein-driven melanosomes (results not shown). The uncertainty on the particle position determination makes the PDDF broader and, as a consequence, the value of κ is underestimated as will be further discussed below. Jin et al [32] showed that this error does not affect the shape of the PDDF and thus the confinement mechanism can be clearly distinguish by this analysis. Unfortunately, these calculations require the simultaneous analysis of multiple trajectories and thus they only provide the average behavior of the organelles.

On the other hand, Wang et al [33] studied the motion along microtubules of different proteins -including dynein and kinesin- attached to beads in *in vitr*o assays. They found that the distribution of axial positions follows a Gaussian function for proteins that move or are attached to a single protofilament while those proteins that switch protofilaments present a more flat distribution with multiple peaks. Thus, the Gaussian distribution observed in Figure 2 suggests that melanosomes do not switch protofilaments in the studied time window.

### Melanosomes microenvironment is viscoelastic

The initial slope of the MSD_⊥_ vs τ plot is also related to the rheological properties of the microenvironment, e.g. we would expect a fast increment of the MSD_⊥_ for an aqueous microenvironment while for a high viscous liquid the saturation is obtained at higher time lags. Moreover, if the microenvironment is purely viscous MSD_⊥_ would reach the plateau exponentially. The viscoelasticity of the surrounding medium affects the dynamics of the transported organelle in a complex way, determining a non-exponential behavior in the approach to the plateau region [33].

To obtain information regarding the rheological properties of the medium, we analyzed the power spectrum density (PSD) of the 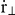 data. This approach is widely used in optical trap experiments, either to calibrate the stiffness of the trap or to determine the rheological behavior of the medium [34], [35], [36], [37]. The analysis was performed for frequencies higher than 13 Hz (i.e. τ <70 ms) since this is the range in which the motion of the organelle is mainly given by the rheological properties of its microenvironment.

The power spectrum in the limit of higher frequencies is characterized by 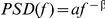 where a is a constant and β  = 2 in the case of a pure viscous fluid [34] while in a viscoelastic medium where the organelle displays a subdiffusive behavior the power spectrum deviates leading to a power law with 1< β < 2 [35], [36], [37].

**Figure 3:**
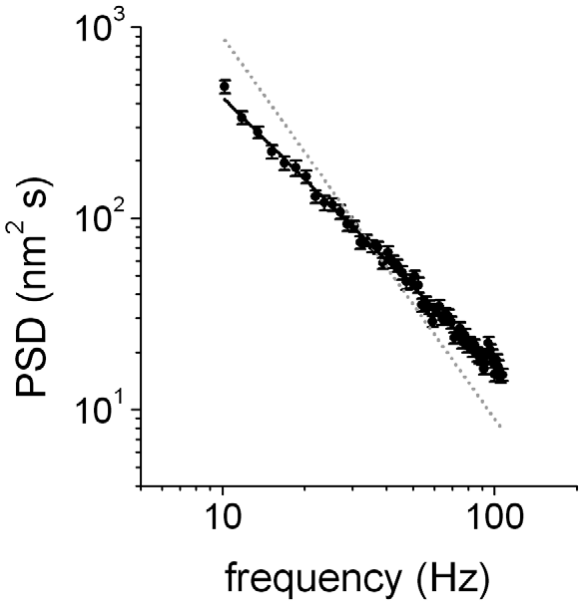
Power spectrum distribution of 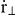
 **.** 260 trajectory segments obtained for dynein-driven organelles during dispersion were analyzed as described to obtain the PSD. The continuous line corresponds to the fitting of PSD  =  af^−β^ with β = 1.41±0.02. Dotted gray line represents the behavior expected for a pure viscous microenvironment.

Figure 3 shows that the PSD of the 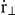 data followed a power law behavior with β  = 1.41±0.02; similar values for the exponents were obtained for kinesin and dynein in every stimulated condition. Therefore, we concluded that the organelle microenvironment is viscoelastic [36], [37], [38], [39], [40].

### Modeling the dynamics of melanosomes

**Figure 4:**
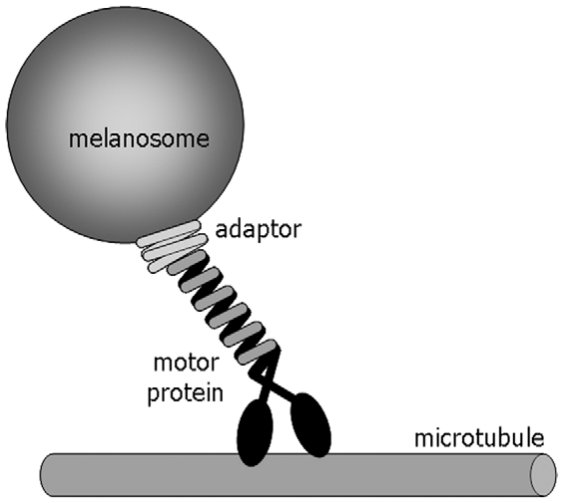
Scheme of the mechanical interaction between organelles and microtubules. To simplify the scheme, a single copy of the motor linker complex is represented.

We assumed a model in which the organelle is attached to the microtubule through a spring-like linker while being transported (Figure 4).

Physically, the linker is constituted by the motor and other molecules that mediate its attachment to the organelle. Each of these molecules have an intrinsic elasticity κ_i_ and therefore, the anchor molecules behave as serial springs with an effective elastic constant κ_ML_ given by κ_ML_^−1^ =  ∑κ_i_^−1^, with the softest element dominating the overall elastic response. Since melanosomes are stiff organelles [41], they do not contribute to the elasticity of the system. In the case of multiple motors linking the organelle to the microtubule, the effective elastic constant will be given by the sum of the elastic constants of each of the motor-anchor protein complex interacting with the organelle. The influence of dynactin in κ_ML_ is more complex since it has both microtubule and motor binding domains [26]. In the following sections, we will refer to the molecules involved in binding the organelle to the microtubule as the *motor linker*.

It is important to mention that there are probably other unknown contributions to the linker stiffness such as the diffusion of the motors/adaptor along the membrane of the organelle and the attachment/detachment of motors which may be contributing to the transport.

On the other hand, the organelle also experiences a drag force as it moves in the extremely crowded intracellular medium. The organelle motion perpendicular to the transport direction axis can be described by a generalized Langevin equation,
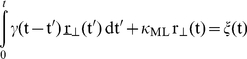
(4)where the inertial contribution was neglected.

Equation (4) describes the dynamics of a particle in a harmonic well under overdamped (high friction) conditions, and immersed in a viscoelastic environment. 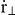 represents the instantaneous velocity of the organelle in the perpendicular direction and γ(t) is the time-dependent frictional coefficient that characterizes the viscoelastic properties of the cytoplasm. In a pure viscous environment, this term reduces to the well known Stokes drag force 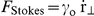.

The second term corresponds to the spring-like force, being κ_ML_ the effective trapping stiffness of the motor linker in the direction perpendicular to the active motion. Finally, the random force *ξ*(t) represents the internal noise due to thermal activity. It is a zero-centered and stationary function with correlation function 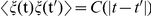, and is related to the time-dependent frictional coefficient γ(t) via the fluctuation-dissipation theorem [42], 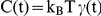.

A detailed analysis of equation (4) can be found in [43], [44]. In particular, MSD_⊥_ can be expressed as,
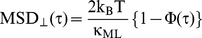
(5)where Φ(τ) is the inverse Laplace transform of the kernel,
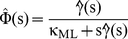
(6)


Considering that 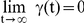, it can be demonstrated that [43], [44]

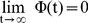
(7)


Then, the saturation value of the MSD_⊥_ is given by [44],
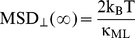
(8)


This expression shows that MSD_⊥_(∞) depends only on the spring constant and the temperature. Then, analysis of the MSD in the asymptotic regime provides a valuable tool to determine the stiffness of the motor linker, independently of the viscoelastic properties of the medium which are related to the behavior of the Φ function.

To compare the analytical expression with the experimental data it is necessary to take into account the error on the particle localization. This error can be included as an uncorrelated noise of variance δ^2^ [45], [46] and thus equation (8) is modified as follows,
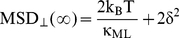
(9)


### Organelles attach to microtubules through linkers with low stiffness

We analyzed the MSD_⊥_ data obtained before and calculated the value of κ_ML_ for every trajectory segment obtained in aggregation or dispersion conditions by using equation (9). To have a precise measurement of the experimental noise δ in every experiment, we measured the error on the position determination of melanosomes in formaldehyde-fixed cells before and after the experiment. Also, we measure δ by linear extrapolation to τ  = 0 of the first 5 points of each MSD_⊥_ vs τ curve. The values obtained from these extrapolations were identical to those obtained in the calibration experiments with fixed cells showing that both methods for δ determination were equally good.

**Figure 5:**
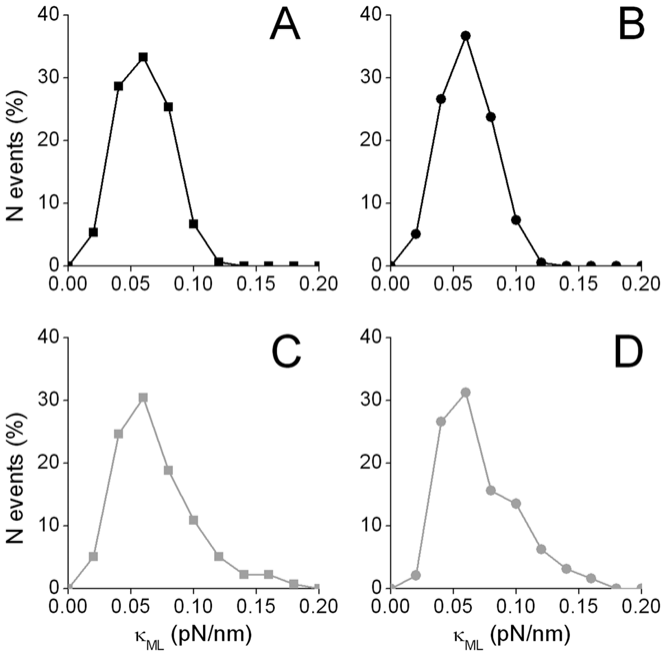
Distributions of κ_ML_ for motor-attached melanosomes. The values for the stiffness of kinesin (squares) and dynein (circles) driven melanosomes were obtained as described in the text during dispersion (gray symbols) and aggregation (black symbols). Each histogram includes data from 150 to 200 trajectory segments, depending on the condition.

Figure 5 shows that κ_ML_ ranged between 0.02–0.2 pN/nm with most probable values of 0.0594±0.0005 and 0.059±0.001 pN/nm (dynein and kinesin during aggregation, respectively) and, 0.060±0.003 and 0.060±0.002 pN/nm (dynein and kinesin during dispersion, respectively).

As we mentioned before, the value of κ_ML_ is underestimated in the PDDF analysis (Figure 2). Jin et al [32] simulated trajectories of particles in a spring-like potential and added to the trajectories a random noise corresponding to the uncertainty on the particle position determination which was sampled from normal distributions with zero mean and standard deviation δ. They verified that the estimated value of κ decreases with the ratio δ/r_c_ where r_c_ is the confinement radius. We estimated the confinement radius of melanosomes from MSD_⊥_(∞)  =  r_c_^2^ [32], calculated the ratio δ/r_c_ and derived from the data presented by Jin et al [32] that κ_ML_ obtained by PDDF analysis is underestimated in ∼40% in our experimental conditions. Thus the corrected stiffness is ∼0.05 pN/nm agreeing well with the data obtained from the analysis of the MSD_⊥_.

Our results indicate that the stiffness of the motor linker in living cells is about one order lower than those reported for kinesin in *in vitro* conditions [17], [18], [19], [20] probably due to the fact that the anchoring of motors to the organelles provides additional flexibility to the motor linker.

Interestingly, the distributions and characteristic values of κ_ML_ measured for dynein and kinesin motors were not significantly different in the assayed conditions pointing toward a similar weak, mechanical coupling between the organelle and the microtubule when the linker is either of these motors.

Figure 5 also shows that κ_ML_ for dynein and kinesin driven melanosomes during dispersion present tailed distributions, not observed during aggregation. Future studies will be needed to explore the molecular mechanisms determining these different behaviors.

### The stiffness of the linker depends on dynactin integrity

We mentioned above that the protein complex dynactin anchors dynein and kinesin motors to the membrane of melanosomes [27] and thus has a key regulatory role on the microtubule-dependent transport properties. Therefore, we hypothesize that disruption of this complex might affect the MSD behavior studied before.

In order to test this hypothesis, we studied the dynamics of melanosomes in cells overexpressing the dynactin subunit dynamitin (p50) fused to EGFP. This treatment promotes the dissociation of the motor-binding domain of dynactin from the cargo-binding domain [47], [48], [49]. As a consequence, both plus and minus end directed transport of organelles such as melanosomes [27] and endosomes [28] are drastically reduced.

**Figure 6:**
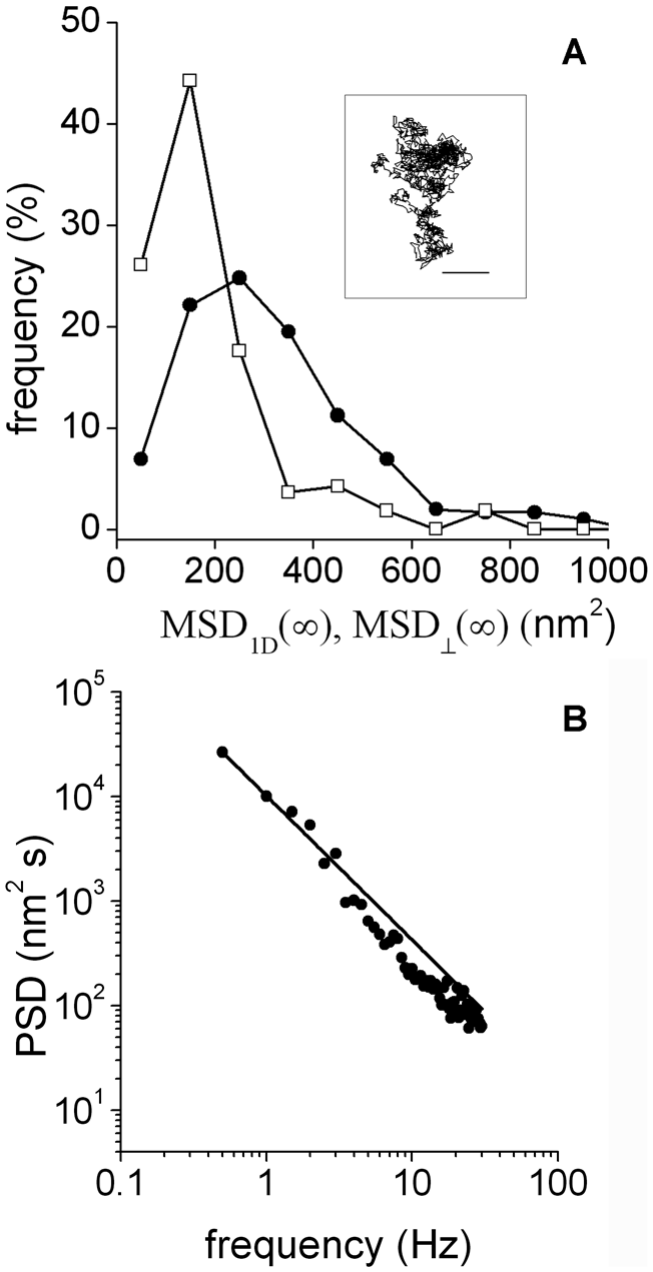
Effects of p50 overexpression on melanosome dynamics. (A) Distribution of MSD_1D_(∞) in p50 overexpressing cells (circles). The histogram was constructed with data from 304 trajectory segments as described in the text. The distribution of MSD_⊥_(∞) for melanosomes driven by dynein during aggregation (squares) is also represented to make the comparison of these data easier. In both cases, the term corresponding to the error on the melanosome position determination was subtracted. Inset: representative trajectory of a melanosome in a p50 overexpressing cell. Scale bar, 100 nm. (B) Power spectrum distribution. 260 trajectory segments were analyzed as described before to obtain the PSD of either the x or y coordinate of the melanosomes. The continuous line corresponds to the fitting of a power-law behavior with β = 1.38±0.02.

We verified that the number of organelles actively transported strongly decreases in melanophores overexpressing p50 as was previously observed by Deacon et al [27]. Agreeing with this qualitative observation, we registered trajectories of melanosomes and observed that most of the organelles displayed confined-like or anomalous subdiffusion (Inset to Figure 6A) as assessed by MSD analysis. This last behavior was expected since motion of inert particles in the cytoplasm is influenced by other processes such as the remodeling and reorganization of the cytoskeleton [50]. In these cells, the average distance traveled by melanosomes after ∼75 ms (i.e. the time lag in which MSD_⊥_ reaches the constant value in wild type cells) is ∼320 nm.

We divided melanosome trajectories in segments of 200 data points and only analyzed those presenting confined motion since other segments are probably influenced by the active processes discussed before. To make the comparison between P50-overexpressing and wild type cells easier we analyzed the behavior of the MSD obtained for either the x or y coordinates (MSD_1D_) after subtracting the term corresponding to the error on the particle localization (2δ^2^ in equation 9). Figure 6A showed that MSD_1D_ (∞) -i.e. the value of MSD_1D_ at the plateu is ∼250 nm^2^, which is significantly larger than MSD_⊥_(∞) determined in wild type cells (∼130 nm^2^).

These results indicate that organelles not actively transported by motors in p50 overexpressing cells are confined to a larger region than organelles linked to microtubules in wild type cells supporting the fact that MSD_⊥_(∞) in wild type cells is given by the stiffness of the motor linker.

We also performed a PSD analysis on these trajectories (Figure 6B) and observed that the microenvironment to the organelles is viscoelastic with β = 1.38±0.02. This value is not significantly different from that measured in wild type cells indicating that the organelles are sensing similar microenvironments.

We also analyzed the trajectories of the few melanosomes that presented motion compatible with active transport in p50 overexpressing cells and verified that these organelles present stiffness values similar to those observed in wild type cells (not shown). This result supported that the overexpression is probably not fully efficient and thus a small number of organelles are still actively transported.

### Final Remarks

In this work, we used single particle tracking to characterize the stiffness of the complex constituted by active motors and molecules mediating their attachment to melanosomes in living cells. We tracked these organelles with high temporal and spatial resolutions and characterized their dynamics perpendicular to the cytoskeleton track. This motion was due to the spring-like interaction between melanosomes and microtubules in a viscoelastic microenvironment. Our results indicate that the stiffness of the motor linker in living cells is about one order lower than that reported for kinesin in *in vitro* conditions [17], [18], [19], [20]. This different behavior is probably due to the fact that the adaptor molecules mediating the anchoring of motors to the organelles provides additional flexibility pointing out the relevance of performing these determinations in the cellular context. As we mentioned before, the dynamical organization of motors within the membrane of melanosomes is not completely known and thus it is not possible to evaluate how this organization influences the overall stiffness.

It was recently demonstrated that the stiffness of the motor plays an important role when multiple motors collectively transport a cargo, which is a typical situation during organelle transport in live cells (reviewed in [51]). In a recent paper, Bieling et al [52] showed that constructs of kinesin-1 lacking most of the non-motor and potentially flexible regions interfere with each other when transporting a microtubule in sliding assays, reducing the speed of the transported microtubule. Interestingly, the properties of this construct at the single molecule level were identical to those of the full-length kinesin-1. These authors suggest that these non-motor regions are essential for a loose mechanical coupling between motors. In this condition, the attached motors do not generate a high counterforce when one of the motors stochastically steps on the microtubule.

In this context, the low stiffness determined in our work for the motor linker would reflect the requirement of loose mechanical coupling necessary to prevent the interference of motors during collective transport of organelles in live cells. We are currently working on a stochastic model to describe the coordination of motors of same or different polarity which takes into account the low stiffness determined in this work for kinesin and dynein driven melanosomes.

## Materials and Methods

### Cell culture and samples preparation for imaging

Immortalized *Xenopus laevis* melanophores were cultured as described in [53]. In order to track the movement of individual organelles, the number of melanosomes in cells was reduced by treatment with phenylthiourea [54].

For microscopy measurements, cells were grown for 2 days on 25-mm round polylysine-coated coverslips placed into 35-mm plates in 2.5 ml of the medium. Before observation, the coverslips were washed in serum-free 70% L-15 medium and mounted in a custom-made chamber specially designed for the microscope. The cells were treated with 10 µM latrunculin B (Biomol International, Plymouth Meeting, PA) for at least 30 min to depolymerize actin filaments. Melanophores were stimulated for aggregation or dispersion with 10 nM melatonin or 100 nM MSH, respectively. Samples were observed between 5 to 20 min after stimulation. All measurements were performed at 21°C. Microtubule tracking experiments were done using a cell line of melanophores stably expressing EGFP-tagged XTP, a *Xenopus* homologue of tau protein which bind to microtubules and allows their visualization in live cells [10], [55]. This cell line is a kind gift of Dr. Vladimir I Gelfand (Northwestern University, Chicago, IL).

pEGFP–dynamitin was a kind gift from Dr. Vladimir Gelfand (Northwestern University, Chicago, IL). Melanophores were transfected using the FuGENE6 transfection reagent (Roche Diagnostics).

### Tracking experiments

Single particle tracking experiments of melanosomes moving along microtubules in wild type cells were carried out in a FV1000 microscope adapted for SPT using a 60x oil-immersion objective (numerical aperture  = 1.25) under illumination with a tungsten-halogen lamp. A high-speed electron-multiplying CCD camera (Cascade 128+, Photometrics, Tucson, AZ) was attached to the video port of the microscope for imaging the cells. 2000-frames movies were registered at speeds of 330 or 100 frames/s depending on the experiment. Trajectories of melanosomes were recovered from these movies using the pattern-recognition algorithm described in [10]. The accuracy on the melanosome position determination was in the range 4–7 nm.

Since the algorithm does not allow detecting rotation of the particle which would introduce an additional error in case of asymmetric particles, we never analyze the trajectories of asymmetric objects (e.g. two melanosomes in direct contact or close to each other). On the other hand single melanosomes can be considered spherical in our imaging conditions as assessed by analyzing the intensity distribution of images of the organelles.

Tracking experiments of microtubules were done in the confocal microscope described before. The excitation source was a multi-line Ar laser tuned at 488 nm (average power at the sample, 700 nW). The laser light was reflected by a dichroic mirror (DM405/488) and focused through an Olympus UPlanSApo 60x oil immersion objective (NA = 1.35) onto the sample. The fluorescence was collected by the same objective, passed through the pinhole, reflected on a diffraction grating, and passed through a slit set to transmit in the range 500–600 nm. Fluorescence was detected by a photomultiplier set in the photon-counting detection mode. The pixel size was set to 41 nm.

### Data analysis

The power spectrum density (PSD) was obtained by computing the discrete Fourier Transform of the time course of the position of either melanosomes or microtubules and calculated as 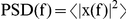 were x is the position of the particle under study and f is the frequency. Typically, segments of 200 data points obtained with a sampling frequency of 330 or 500 Hz (melanosomes) and 170–250 Hz (microtubules after binning 2 continuous scanning lines) were analyzed and averaged. These analyses were performed using Matlab standard routines.

Histograms were constructed setting the bin size to the value determined by [56]:
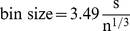
(10)


where n is the number of data and s is an estimate of the standard deviation.

## Supporting Information

Figure S1 **Tracking of microtubules by confocal imaging.** (A) Image of a region of a melanophore cell expressing XTP-GFP. The line scanned by the laser beam is showed in yellow. Scale bar, 2 µm. The right panel showed the intensity matrix obtained in a representative tracking experiment. Intensity values are displayed in pseudocolor. Each row of this matrix corresponds to one scanning line and each column shows the time evolution of the intensity at a given pixel. The lateral displacement of the microtubule as a function of time was recovered from this data as described in the text for experiments with signal/noise ratio >20. (B) Normalized PSD obtained by Fast Fourier transform of microtubule trajectories in living cells and analyzed as described in the text.
(TIF)

Figure S2 **Fluctuations on microtubule fluorescence intensity.** (A) Representative example of the intensity fluctuations observed at the microtubule during the tracking experiment. (B) Dependence of the accuracy on the particle position determination (σ) as a function of the intensity of the particle (I). The data was obtained as described in the text and fitted with a function σ  =  a I^b^ with the best-fitting parameters a  =  500 ± 200 nm and b  =  -0.78 ± 0.08 (continuous line). (C) Power spectrum distribution obtained from the simulation of fixed microtubules with fluctuating intensity (described in the text).
(TIF)

Text S1 **Microtubule motion do not contribute to the dynamics of the organelles in the studied temporal window.**
(DOC)
