# Supplementary figures and images for: Mechanical Properties of Organelles Driven by Microtubule-Dependent Molecular Motors in Living Cells
Source: PLoS One. 2011 Apr 1;6(4):e18332. doi: 10.1371/journal.pone.0018332 (PMC3069964; doi:10.1371/journal.pone.0018332)

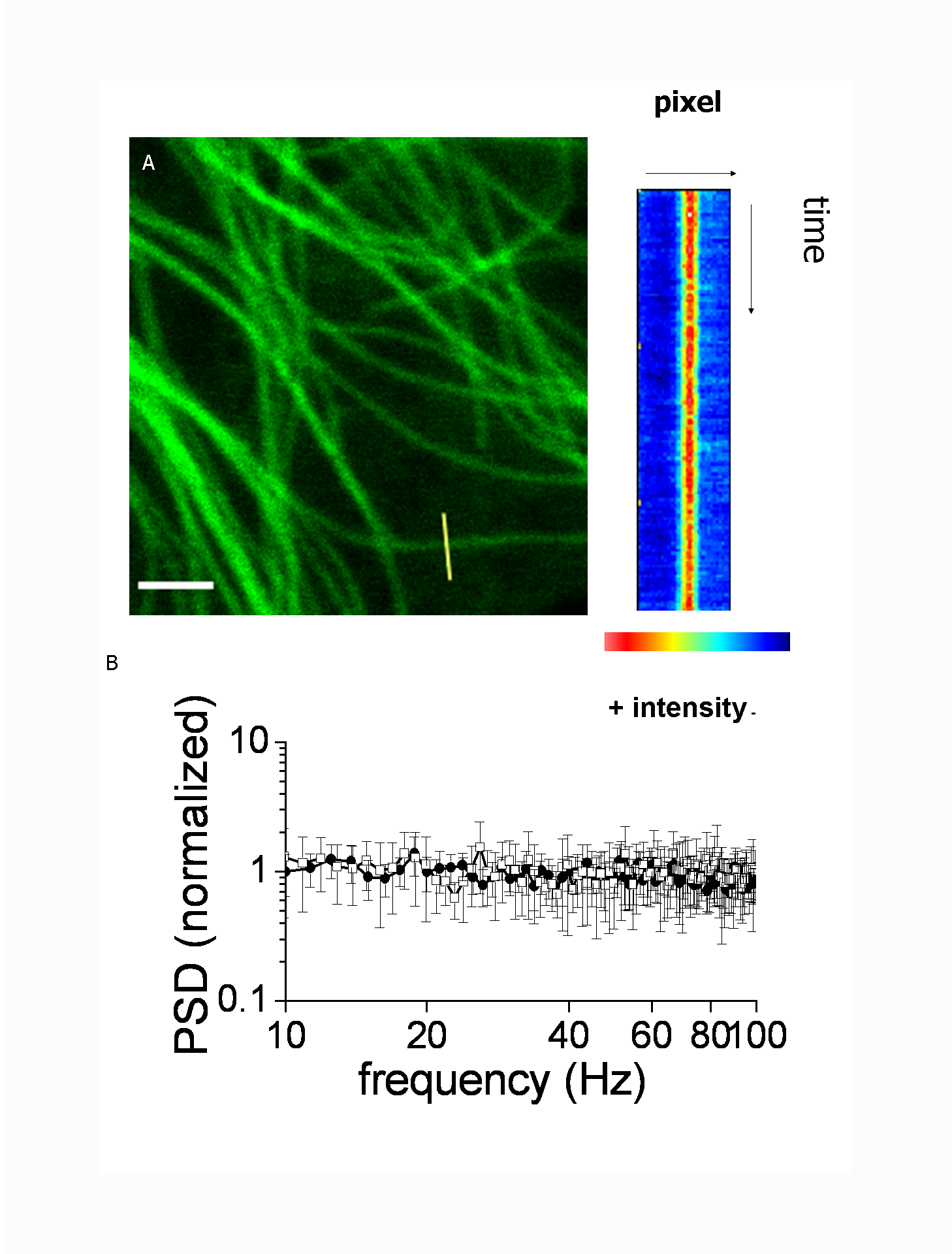

Supplement: Figure S1 — Tracking of microtubules by confocal imaging. (A) Image of a region of a melanophore cell expressing XTP-GFP. The line scanned by the laser beam is showed in yellow. Scale bar, 2 µm. The right panel showed the intensity matrix obtained in a representative tracking experiment. Intensity values are displayed in pseudocolor. Each row of this matrix corresponds to one scanning line and each column shows the time evolution of the intensity at a given pixel. The lateral displacement of the microtubule as a function of time was recovered from this data as described in the text for experiments with signal/noise ratio >20. (B) Normalized PSD obtained by Fast Fourier transform of microtubule trajectories in living cells and analyzed as described in the text. (TIF) [file pone.0018332.s001.tif]

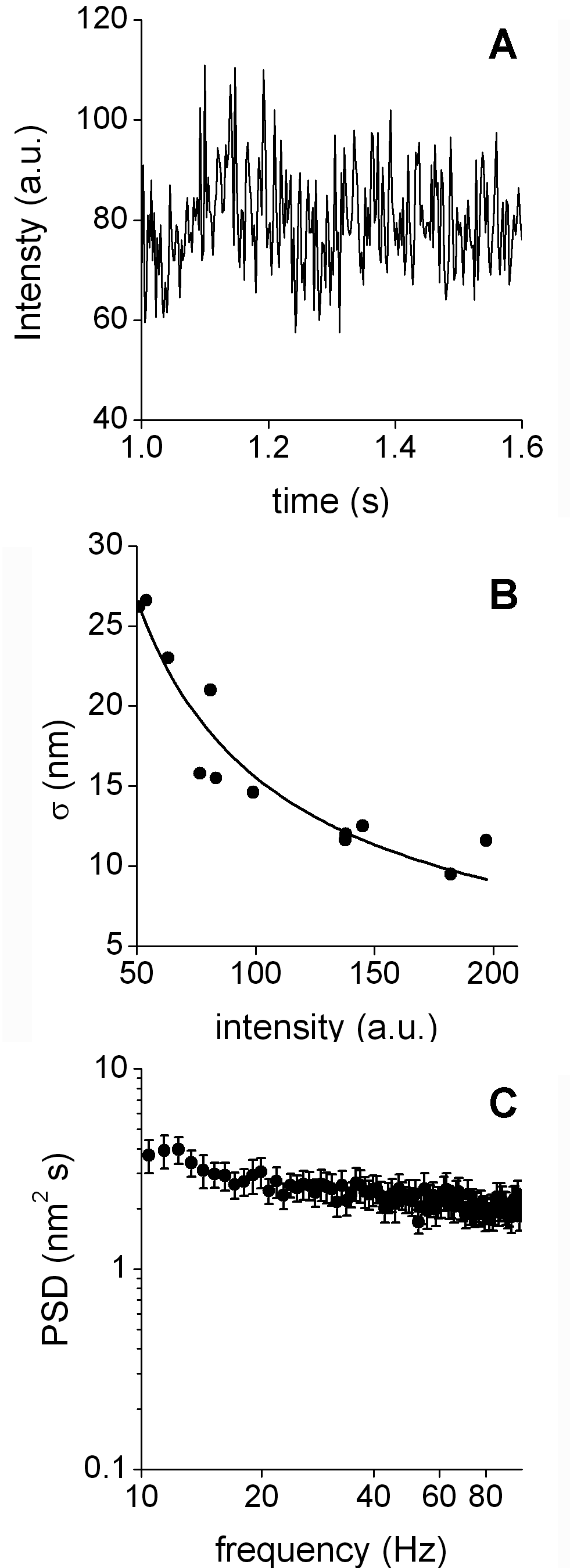

Supplement: Figure S2 — Fluctuations on microtubule fluorescence intensity. (A) Representative example of the intensity fluctuations observed at the microtubule during the tracking experiment. (B) Dependence of the accuracy on the particle position determination (σ) as a function of the intensity of the particle (I). The data was obtained as described in the text and fitted with a function σ = a Ib with the best-fitting parameters a = 500 ± 200 nm and b = -0.78 ± 0.08 (continuous line). (C) Power spectrum distribution obtained from the simulation of fixed microtubules with fluctuating intensity (described in the text). (TIF) [file pone.0018332.s002.tif]
