# Supplementary material for: Mechanical Properties of Organelles Driven by Microtubule-Dependent Molecular Motors in Living Cells
Source: PLoS One. 2011 Apr 1;6(4):e18332. doi: 10.1371/journal.pone.0018332 (PMC3069964; doi:10.1371/journal.pone.0018332)
Supplement: Text S1 — Microtubule motion do not contribute to the dynamics of the organelles in the studied temporal window. (DOC) [file pone.0018332.s003.doc]

**Supporting text.** **Microtubule motion do not contribute to the dynamics of the organelles in the studied temporal window**

In order to characterize the movement of the microtubule within the temporal window of the melanosome tracking experiments (i.e., 0.6 s), we followed the motion of microtubules in melanophore cells expressing EGFP-tagged XTP by using a confocal microscope. In these experiments the laser is set to scan a line perpendicular to a microtubule in a repetitive fashion (Figure S1A). The position of the microtubule intersected by the laser is then calculated by fitting a Gaussian function to the intensity profile obtained after binning two continuous lines. This procedure allows us to track the microtubule in 4-5 ms, i.e. similar to the temporal resolution of the tracking experiments of melanosomes, with an accuracy of ~30 nm as assessed in fixed samples. Since the noise of these determinations is high, we analyzed the fluctuations on the position of microtubules by calculating the power spectrum distribution (PSD) of these trajectories (Fig S1B).

Fig S2A shows that the intensity at microtubule positions present high fluctuations probably related to the binding/unbinding of XTP-GFP. Since the accuracy on the position determination is intimately related to the fluorescence intensity collected for the particle we analyzed if these fluctuations affect the behavior of the PSD.

With this aim, we run a control experiment in which we tracked fixed particles with different brightnesses using the tracking procedure employed to follow microtubules. Figure S2B shows that the error on the particle position determination significantly decreases as a function of the average intensity collected in each line scan.

Therefore, we can consider that

$x_{m}(t)=x(t)+\varsigma(t)$ (S1)

where x_m_ and x are the measured and real positions of the microtubule, respectivley and ς is a zero-centered function representing the error on the particle position. This error is given by the fluorescence intensity (Fig S2B) and is uncorrelated with the actual microtubule position.

The total PSD of the position then becomes ,

$\left\langle\left|x_{m}(f)\right|^{2}\right\rangle=\left\langle\left|x(f)\right|^{2}\right\rangle+\left\langle\left|\varsigma(f)\right|^{2}\right\rangle$ (S2)

In order to determine $\varsigma(t)$ for the experiments performed in this section we analyzed the intensity at each experimental data point and assigned the expected value for the error using the calibration curve showed before. Figure S2C shows that the PSD of this data presents a slight dependence with the frequency showing that intensity fluctuations introduce an artifact in the PSD calculation.

To take into account this error, we analyzed the ratio between the PSD of microtubules and that of Figure S2C. Figure S1B shows that this ratio does not depend on the frequency and is not significantly different from the behavior observed for microtubules in fixed cells. This result indicates that the PSD of microtubules do not have other spectral contributions different from those introduced by the intensity fluctuations.

Therefore, we can conclude that the microtubule tracks remain stationary within the 600 ms-time window in which we study the dynamics of melanosomes. This result agrees with those presented by Kural et al also supporting that microtubules move much slower than organelles.

Supporting references

1. Thompson RE, Larson DR, Webb WW (2002) Precise nanometer localization analysis for individual fluorescent probes. Biophys J 82: 2775-2783.

2. Savin T, Doyle PS (2005) Static and dynamic errors in particle tracking microrheology. Biophys J 88: 623-638.

3. Kural C, Kim H, Syed S, Goshima G, Gelfand VI, et al. (2005) Kinesin and Dynein Move a Peroxisome in Vivo: A Tug-of-War or Coordinated Movement? Science 308: 1469-1472.
